# Complex N-Linked Glycosylation: A Potential Modifier of Niemann–Pick Disease, Type C1 Pathology

**DOI:** 10.3390/ijms23095082

**Published:** 2022-05-03

**Authors:** Niamh X. Cawley, Anna T. Lyons, Daniel Abebe, Rachel Luke, Julia Yerger, Rebecca Telese, Christopher A. Wassif, Joan E. Bailey-Wilson, Forbes D. Porter

**Affiliations:** 1 Division of Translational Medicine, Eunice Kennedy Shriver National Institute of Child Health and Human Development, National Institutes of Health, Bethesda, MD 20892, USA; cawleyn@mail.nih.gov (N.X.C.); atlyons@mcw.edu (A.T.L.); rachel.luke@nih.gov (R.L.); julia.yerger28@gmail.com (J.Y.); teleres@acom.edu (R.T.); wassifc@cc1.nichd.nih.gov (C.A.W.); 2 Research Animal Management Branch, Eunice Kennedy Shriver National Institute of Child Health and Human Development, National Institutes of Health, Department of Health and Human Services, Bethesda, MD 20892, USA; abebed@mail.nih.gov; 3 Computational and Statistical Genomics Branch, National Human Genome Research Institute, National Institutes of Health, Department of Health and Human Services, Baltimore, MD 21224, USA; jebw@mail.nih.gov

**Keywords:** Niemann–Pick disease, type C, N-linked glycosylation, MGAT5, LAMP1, disease severity score, Purkinje neuron

## Abstract

Complex asparagine-linked glycosylation plays key roles in cellular functions, including cellular signaling, protein stability, and immune response. Previously, we characterized the appearance of a complex asparagine-linked glycosylated form of lysosome-associated membrane protein 1 (LAMP1) in the cerebellum of *Npc1^−/−^* mice. This LAMP1 form was found on activated microglia, and its appearance correlated both spatially and temporally with cerebellar Purkinje neuron loss. To test the importance of complex asparagine-linked glycosylation in NPC1 pathology, we generated NPC1 knock-out mice deficient in MGAT5, a key Golgi-resident glycosyl transferase involved in complex asparagine-linked glycosylation. Our results show that *Mgat5^−/−^:Npc1^−/−^* mice were smaller than *Mgat5^+/+^:Npc1^−/−^* mice, and exhibited earlier NPC1 disease onset and reduced lifespan. Western blot and lectin binding analyses of cerebellar extracts confirmed the reduction in complex asparagine-linked glycosylation, and the absence of the hyper-glycosylated LAMP1 previously observed. Western blot analysis of cerebellar extracts demonstrated reduced calbindin staining in *Mgat5^−/−^:Npc1^−/−^* mice compared to *Mgat5^+/+^:Npc1^−/−^* mutant mice, and immunofluorescent staining of cerebellar sections indicated decreased levels of Purkinje neurons and increased astrogliosis in *Mgat5^−/−^:Npc1^−/−^* mice. Our results suggest that reduced asparagine-linked glycosylation increases NPC1 disease severity in mice, and leads to the hypothesis that mutations in genes involved in asparagine-linked glycosylation may contribute to disease severity progression in individuals with NPC1. To examine this with respect to *MGAT5,* we analyzed 111 NPC1 patients for two *MGAT5* SNPs associated with multiple sclerosis; however, we did not identify an association with NPC1 phenotypic severity.

## 1. Introduction

Niemann–Pick disease type C (NPC) is a fatal, neurodegenerative lysosomal disorder. It is inherited in an autosomal recessive manner and occurs with an estimated incidence of 1:100,000 to 1:150,000 births [1,2]. Patients have pathological variants in either *NPC1* (95% of cases) or *NPC2*, which code for proteins involved in the binding and efflux of unesterified cholesterol out of the late endosome/lysosome compartments [3,4]. Pathological variants in either gene results in the accumulation of unesterified cholesterol and glycosphingolipids within the late endosomal/lysosomal system [5,6]. This eventually leads to cellular dysfunction, and in the case of the cerebellum, to the degeneration of Purkinje neurons [7]. The clinical phenotype of this disease is marked by hepatosplenomegaly, cerebellar ataxia, and progressive cognitive decline [1]. Early death of the patient ultimately occurs [8].

As a key organelle in the progression of NPC, the integrity and function of the lysosome, and its limiting membrane, is critical. The lysosomal membrane is of particular interest, where the complexity and integrity of the ~8 nm glycocalyx [9] is important since increased lysosomal membrane permeability has been shown to occur in microglia, resulting in the leakage of hydrolytic enzymes, such as cathepsin B, and cell death [10]. Heat shock protein 70 (HSP70) has been shown to stabilize lysosomal membrane permeability as part of its pro-survival mechanism [11,12]. Thus, Arimoclomol^TM^, a drug currently undergoing clinical trials for treatment of NPC1, which induces HSP70, is proposed to stabilize the lysosomal membrane in NPC1 as part of its mechanism of action. Lysosome-associated membrane protein 1 and 2 (LAMP1/2), two major proteins of the lysosomal membrane [13,14], are heavily glycosylated on their luminal domains and contribute to the glycocalyx and integrity of the membrane. This helps form a barrier between the hydrolytic enzymes in the lumen of the lysosome and its lipid bilayer. Hence, the integrity and structure of the glycocalyx may modulate neuronal survival in NPC1.

The study of protein glycosylation in the context of NPC is limited, where studies have described different glycosylation levels of several proteins, such as NPC2 [15] and ApoE2 [16], reflecting a perturbance of normal glycoprotein homeostasis in NPC with possible functional consequences. Previous studies have analyzed the glycome in cell models of NPC1 disease. In whole cell lysates of Chinese hamster ovary (CHO) cells devoid of NPC1, there was a significant increase in N-glycoproteins overall, with the major increase in high-mannose-type glycoproteins among other glyco-molecules. In addition, complex-type N-glycans were significantly more modified with fucose and sialic acid [17]. Consistent with this, and as a means to analyze the lysosomal glycocalyx, Kosicek et al. [18] undertook a more focused glycomic analysis and comparison between whole cell extracts and purified lysosomal membrane proteins from *Npc1-null* CHO cells. This study revealed a significant increase in sialylated complex N-glycans, specifically in the lysosomal membranes of *Npc1-null* cells [18].

To further investigate the role of complex N-glycosylation in NPC disease, we assessed disease progression in a mouse model of NPC1 where complex N-linked glycosylation was genetically disrupted. We used mice deficient in *Mgat5*, a gene that codes for β1,6N-acetylglucosaminyltransferase V (MGAT5), which is necessary for the biosynthesis of β1,6GlcNAc-branched N-linked glycans [19]. *Mgat5^−/−^* mice are viable, and do not appear to display an obvious mutant phenotype; however, the mice are reported to exhibit an increased immune response in the form of CD4+ T-cell and macrophage activation, as well as decreased tumor metastasis, in models of cancer metastasis [20,21]. In our studies, we found that mice null for both *Npc1* and *Mgat5* (*Mgat5^−/−^:Npc1^−/−^*) were more severely affected than *Npc1^−/−^* mice in terms of phenotypic scoring metrics, beam walk latency, and biochemical markers of NPC1 disease. The mice also had a shorter lifespan. Our results suggest that disruption of the complex N-glycosylation pathway is detrimental in NPC1 and suggest that variants in genes involved in the complex N-linked glycosylation pathway may become phenotypic modifiers of NPC1 disease.

## 2. Results

### 2.1. Characterization of Mgat5^−/−^:Npc1^−/−^ Mice

**Figure 1:**
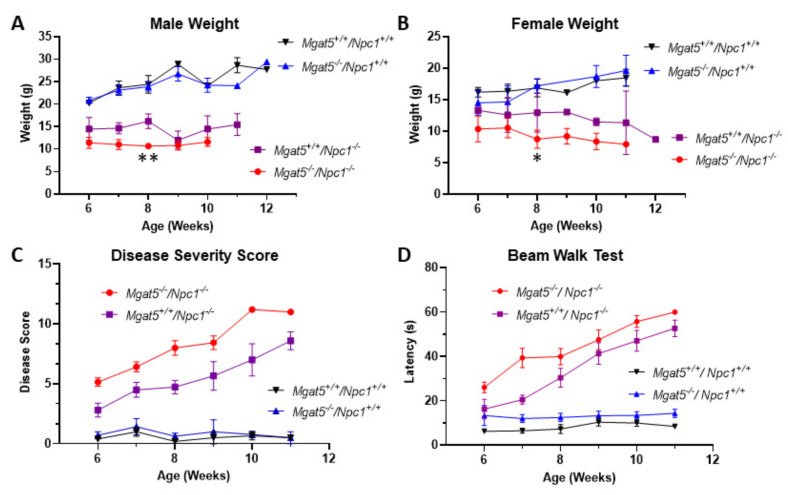
Weight profiles, disease assessment, and beam walk test results of mutant mice. Male (**A**) and female (**B**) mice were weighed weekly starting at week 6 until end-of-life. The weights of *Mgat5^+/+^:Npc1^+/+^*, *Mgat5^−/−^:Npc1^+/+^*, *Mgat5^+/+^:Npc1^−/−^*, and *Mgat5^−/−^:Npc1^−/−^* mice are plotted here. ** *p* < 0.01 for male and * *p* < 0.05 for female *Mgat5^+/+^:Npc1^−/−^* and *Mgat5^−/−^:Npc1^−/−^* mice. (**C**) Mice were assessed weekly for grooming, motor function, kyphosis, hindlimb clasp, and the ledge test, and assigned a combined disease severity score, plotted here, over time. (**D**) Assessment of balance and coordination was performed using the beam walk test. The latency to traverse the beam was recorded weekly, and is plotted here.

Male and female *Mgat5^−/−^:Npc1^−/−^* mice were smaller in size compared to *Mgat5^+/+^:Npc1^−/−^* mice (Figure S1). The double mutant mice (male n = 10; female n = 8) exhibited lower weights in general compared to single mutant *Mgat5^+/+^: Npc1^−/−^* mice (male n = 6; female n = 5), with a significant weight difference seen at 8 weeks of age (Figure 1A,B). *Mgat5^+/+^:Npc1^+/+^* (n = 4 males, 6 females) and *Mgat5^−/−^:Npc1^+/+^* (n = 6 males, 7 females). Control mice exhibited expected weight gain profiles throughout the study, consistent with normal weight profiles of healthy mice.

The *Mgat5^−/−^:Npc1^−/−^* mice (n = 17) developed an NPC disease phenotype similar to that of *Mgat5^+/+^:Npc1^−/−^* mice (n = 11) (Figure 1C) when assessed weekly for grooming, motor function, kyphosis, hindlimb clasp, and ledge test. However, disease progression in the *Mgat5^−/−^:Npc1^−/−^* mice occurred 1–2 weeks earlier than for the *Mgat5^+/+^:Npc1^−/−^* mice. To compliment these observational studies, latency times to complete the beam walk task, a measure of balance and coordination, was recorded weekly. Both *Mgat5^−/−^:Npc1^−/−^* (n = 17) and *Mgat5^+/+^:Npc1^−/−^* (n = 10) mice performed the task with increasing difficulty over time, indicative of disease progression; however, similar to disease severity assessment, impairment occurred 1–2 weeks earlier in the *Mgat5^−/−^:Npc1^−/−^* mice (Figure 1D). *Mgat5^+/+^:Npc1^+/+^* (n = 7) and *Mgat5^−/−^:Npc1^+/+^* (n = 8) control mice did not exhibit difficulty traversing the beam at all ages tested.

**Figure 2:**
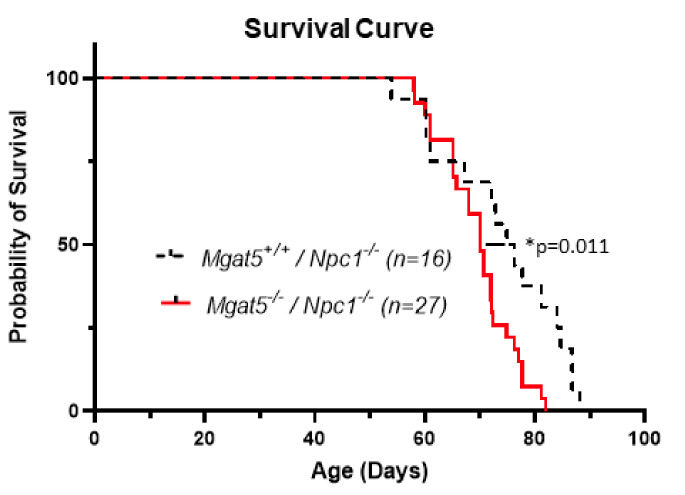
Kaplan–Meier survival curve. Mice were tracked for up to 12 weeks of age and end-of-life recorded. The median age of death of *Mgat5^+/+^:Npc1^−/−^* mice (n = 16) was 75.6 days (range 54–88 days) and the median age of death of *Mgat5^−/−^:Npc1^−/−^* mice (n = 27) was 70 days (range 58–82 days), (log-rank test, * *p* = 0.011).

In survival studies of *Mgat5^−/−^:Npc1^−/−^* mice and *Mgat5^+/+^:Npc1^−/−^* mice, analysis of Kaplan–Meier survival curves showed *Mgat5^−/−^:Npc1^−/−^* mice with a significant reduction in lifespan compared to *Mgat5^+/+^:Npc1^−/−^* mice (Figure 2). The median age of death of *Mgat5^+/+^:Npc1^−/−^* mice (n = 16) was 75.6 days (range 54–88 days) and the median age of death of *Mgat5^−/−^:Npc1^−/−^* mice (n = 27) was 70 days (range 58–82 days) (Mantel–Cox log-rank test, *p* = 0.011).

### 2.2. Western Blot Analysis of Cerebellar Protein from 6-Week-Old Mice

**Figure 3:**
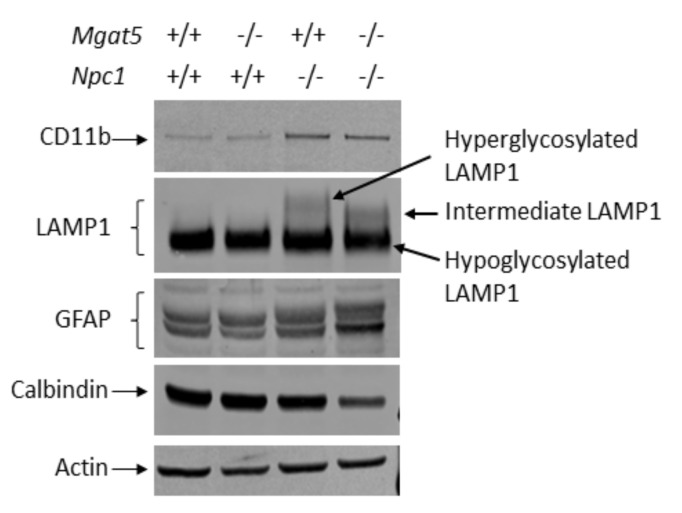
Western blot analysis of 6-week-old cerebellar extracts of mutant mice. Soluble cerebellar protein extracts (40 µg) were analyzed for CD11b (microglia marker), GFAP (astrocyte marker), calbindin (Purkinje neuron marker), and LAMP1 (lysosome marker). In addition to the dominant ~75 kDa hypoglycosylated LAMP1 form present in the cerebellum, hyperglycosylated LAMP1 appears in *Mgat5^+/+^:Npc1^−/−^* mice. In *Mgat5^−/−^:Npc1^−/−^* mice, the hyperglycosylated form of LAMP1 is smaller, giving rise to an intermediate form of LAMP1 lacking complex N-glycosylation. In *Mgat5^−/−^:Npc1^−/−^* mice, GFAP appears increased and calbindin appears decreased relative to *Mgat5^+/+^:Npc1^−/−^* mice, indicative of increased astrogliosis and/or increased Purkinje neuron loss in the double mutant mice. Actin was used as a protein load control. Representative blot of two mice from each genotype.

Higher forms of glycosylated LAMP1 were observed in both *Mgat5^+/+^:Npc1^−/−^* and *Mgat5^−/−^:Npc1^−/−^* mice, as expected from our previous results [22]. The size of the hyperglycosylated LAMP1 from the cerebellum of the *Mgat5^+/+^:Npc1^−/−^* mice was consistent with that previously reported [22]. In the *Mgat5^−/−^:Npc1^−/−^* mice, the size of the upper LAMP1 band was uniformly smaller, consistent with a lack of complex N-linked glycosylation, and is referred to as intermediate LAMP1 (Figure 3). Similar results were seen in liver extracts of affected mice (Figure S2). For cerebellar markers of disease progression, there was an apparent increase in GFAP, a marker of astrogliosis, in both *Mgat5^+/+^:Npc1^−/−^* and *Mgat5^−/−^:Npc1^−/−^* mice; however, the level of GFAP expression appeared higher in the *Mgat5^−/−^:Npc1^−/−^* mice compared to the *Mgat5^+/+^:Npc1^−/−^* mice (Figure 3). CD11b, a marker of microglia, appeared to be increased similarly in both the *Mgat5^+/+^:Npc1^−/−^* and *Mgat5^−/−^:Npc1^−/−^* mice (Figure 3). Calbindin, a Purkinje neuron marker, was analyzed for all genotypes. No significant difference was observed in calbindin levels between *Mgat5^+/+^:Npc1^+/+^* and *Mgat5^−/−^:Npc1^+/+^* control mice (Figure S3A), indicating that the absence of N-linked complex glycosylation alone did not have an effect on Purkinje neuron levels. Likewise, at this age of 5–6 weeks, no difference in calbindin levels were observed between control *Mgat5^+/+^:Npc1^+/+^* and mutant *Mgat5^+/+^:Npc1^−/−^* mice (Figure S3B), consistent with this early “presymptomatic” stage of the disease. However, consistent with disease severity score and beam walk defects, calbindin levels in the double mutant *Mgat5^−/−^:Npc1^−/−^* mice were significantly reduced compared to the *Mgat5^+/+^:Npc1^−/−^* mice at 6 weeks of age (Figure 3 and Figure S3C), consistent with a more severe Purkinje neuron degeneration in the double mutant mice at this age.

### 2.3. Immunohistochemical Analysis of the Cerebellum from 7-Week-Old Mice

**Figure 4:**
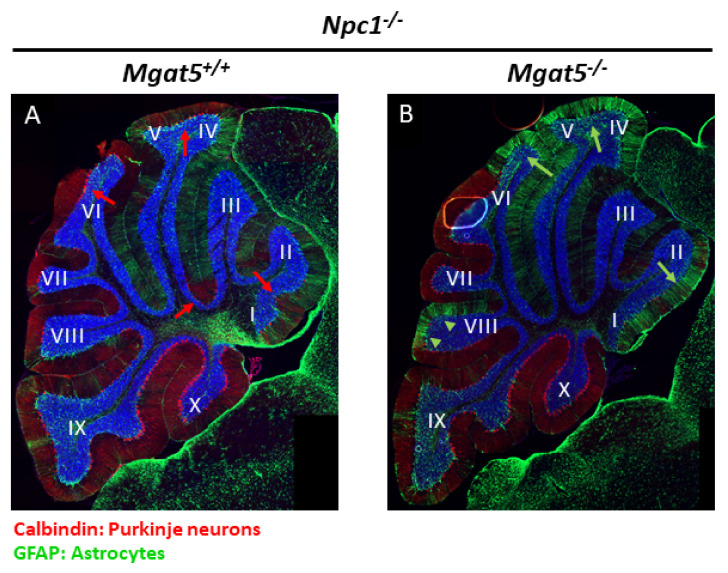
Immunofluorescence analysis of Purkinje neurons and astrocytes in cerebellar sections of mutant mice. The cerebella of mutant mice (7 weeks old) were fixed, sectioned, and stained for GFAP and calbindin. Nuclei were stained with Hoechst. (**A**) In *Mgat5^+/+^:Npc1^−/−^* mice, calbindin staining shows widespread loss of Purkinje neurons in the anterior lobules of the cerebellum, as expected for *Npc1^−/−^* mice at this age. However, some residual Purkinje neurons were observed in the anterior lobules (red arrows). Astrogliosis, as stained by GFAP (green), was also evident in the cerebellum, but appeared more localized to the anterior lobules. (**B**) In *Mgat5^−/−^:Npc1^−/−^* mice, calbindin staining appeared reduced overall, and there were fewer residual Purkinje neurons remaining in the anterior lobules. Astrogliosis appeared more prominent, as stronger GFAP staining was observed in the anterior lobules (green arrows) and some posterior lobules (green arrowheads). Lobules I to X are indicated. Images are representative of one of two sets of mouse cerebella from different breeder lines.

Immunostaining of both *Mgat5^+/+^:Npc1^−/−^* and *Mgat5^−/−^:Npc1^−/−^* mice with anti-GFAP antibodies showed a generalized increase in the staining throughout the cerebella, indicative of astrogliosis (Figure 4, green). The staining of GFAP appeared to extend more into the posterior lobules of the *Mgat5^−/−^:Npc1^−/−^* mice compared to the *Mgat5^+/+^:Npc1^−/−^* mice.

Purkinje neurons, visualized by calbindin immunostaining, were observed in the more posterior lobules of the cerebellum, but were generally absent in the anterior lobules of both *Mgat5^+/+^:Npc1^−/−^* and *Mgat5^−/−^:Npc1^−/−^* mice, as expected (Figure 4, red). However, more Purkinje neurons and their dendritic arbors appeared to be remaining in the anterior lobules of the *Mgat5^+/+^:Npc1^−/−^* mice when compared to the *Mgat5^−/−^:Npc1^−/−^* mice.

**Figure 5:**
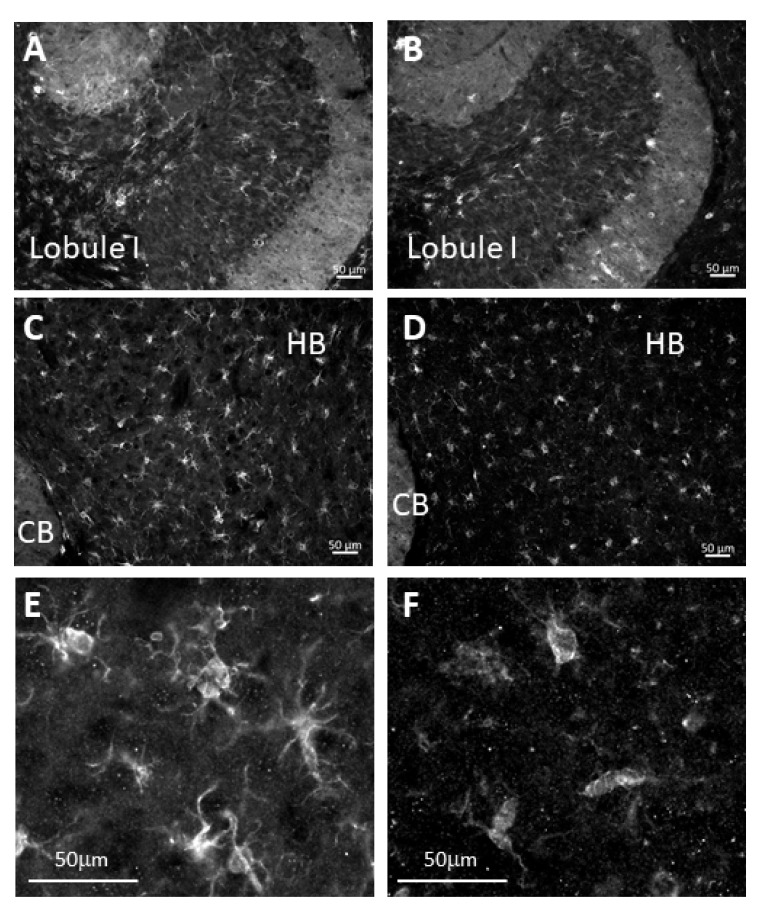
Immunofluorescence analysis of microglia in cerebellar sections of mutant mice. The brains of mutant mice (7 weeks old) were fixed, sectioned, and stained for Iba1. Microglia were observed in the cerebellum (CB) of both *Mgat5^+/+^:Npc1^−/−^* (**A**) and *Mgat5^−/−^:Npc1^−/−^* (**B**) mice; however there were no major differences observed between the lobules (lobule I shown). In the hindbrain (HB) area, adjacent to lobule I of the cerebellum, microglia are observed in both *Mgat5^+/+^:Npc1^−/−^* (**C**) and *Mgat5^−/−^:Npc1^−/−^* (**D**) mice; however, the shape of the microglia were less ramified in the *Mgat5^−/−^:Npc1^−/−^* sections, indicative of a more activated state. Higher magnification of the microglia (Iba1 immunostaining) in this area showed a more ramified shape of the microglia in *Mgat5^+/+^:Npc1^−/−^* mice (**E**) and a more ameboid shape in the *Mgat5^−/−^:Npc1^−/−^* mice (**F**). This indicates a more activated state of microglia in the double mutant mice. Images are representative of one of two sets of mouse cerebella from different breeder lines.

Microglia were visualized by Iba1 immunostaining. Microglia were seen throughout the cerebellar lobules of both *Mgat5^+/+^:Npc1^−/−^* and *Mgat5^−/−^:Npc1^−/−^* mice, an example of which is shown for lobule I (Figure 5A,B). In the grey matter of the hindbrain adjacent to lobule I (Figure 5C,D), the number of microglia appeared equivalent; however, upon analysis at higher magnification, it was seen that the microglia of *Mgat5^+/+^:Npc1^−/−^* mice were ramified in shape, with multiple visible filipodia (Figure 5E). In contrast, the microglia in the *Mgat5^−/−^:Npc1^−/−^* mice lacked the clear appearance of these filipodia (Figure 5F), consistent with the ameboid shape associated with a more activated state of microglia.

### 2.4. Liver Enzyme Levels in Serum

**Figure 6:**
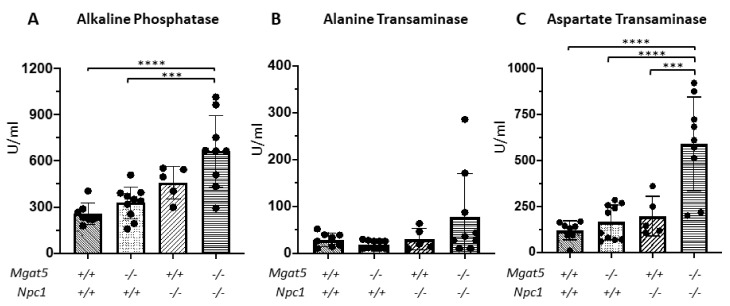
Serum alkaline phosphatase and transaminases. Levels of the liver enzymes, alkaline phosphatase (**A**), alanine transaminase (**B**), and aspartate transaminase (**C**) in the sera of 6-week-old mice were measured. *Mgat5^+/+^:Npc1^+/+^* (n = 8), *Mgat5^−/−^:Npc1^+/+^* (n = 10), *Mgat5^+/+^:Npc1^−/−^* (n = 5), and *Mgat5^−/−^:Npc1^−/−^* (n = 9). *** *p* < 0.001, **** *p* < 0.0001.

As an assessment of liver disease, alkaline phosphatase (AP), alanine transaminase (ALT), and aspartate transaminase (AST) were measured in the serum of 5–6-week-old mice. Both *Mgat5^−/−^:Npc1^−/−^* (n = 9) and *Mgat5^+/+^:Npc1^−/−^* (n = 5) mice exhibited generally elevated levels of these enzymes in their serum compared to the control mice (*Mgat5^+/+^:Npc1^+/+^* (n = 8), and *Mgat5^−/−^:Npc1^+/+^* (n = 10)), suggestive of liver disease in both groups at this age (Figure 6). However, *Mgat5^−/−^:Npc1^−/−^* had significantly higher levels of AST levels compared to *Mgat5^+/+^:Npc1^−/−^* mice, suggestive of a more severe liver disease phenotype.

### 2.5. MGAT5 Single Nucleotide Polymorphism (SNP) Analysis and NPC1 Disease Severity

To investigate the potential association of NPC1 with two human SNPs in *MGAT5,* recently reported to be associated with multiple sclerosis [23], the frequencies of the two *MGAT5* SNPs occurring in 111 NPC1 patient samples were determined. Our analysis shows that the frequencies of the minor alleles for these SNPs are rs4953911 (A = 0.644) and rs3814022 (G = 0.306), and that both SNPs are in Hardy–Weinberg equilibrium (Chi-square (2 df), significance level = 0.958 and 0.971, respectively). In the Caucasian subgroup (81 patients), allele frequency for both SNPs was calculated as rs4953911 (A = 0.636) and rs3814022 (G = 0.327), and was not significantly different than the frequency reported for the European subgroup population (rs4953911 (A = 0.641), rs3814022 (G = 0.268), allele frequency aggregator (ALFA) table, (https://www.ncbi.nlm.nih.gov/snp/, accessed on 20 April 2022), Chi-square (2 df), significance level = 0.969 and 0.530, respectively). This indicates that neither SNP is disproportionately represented in our NPC1 patients. Analysis of age at disease onset, disease severity score at baseline, age-adjusted disease severity score, and age at first neurological symptom, compared to the SNP genotypes in the cohort, did not reveal significant differences between the groups (see Supplementary Figure S4). It is interesting to note that eight patients of Hispanic descent in our cohort had an allele frequency for the A allele at rs4953911 of 0.812, which contrasts with that reported in ALFA of 0.000. This is likely due to the small numbers reported here, and on the NCBI dbSNP website (www.ncbi.nlm.nih.gov/snp/rs4953911, accessed on 20 April 2022). Disease metrics for this group were not significantly different from the rest of the group (data not shown).

## 3. Discussion

Niemann–Pick disease type C1 (NPC1) exhibits marked variability in disease phenotype and onset [24,25], the extent to which is attributed to different *NPC1* mutations that exhibit a more mild or more severe phenotype. It is also due in part to the presence of other factors or disease modifiers that affect disease outcome. These phenotypic modifiers can be environmental factors, genes involved in compensatory pathways, or post-translational modifications. The activation or inhibition of these modifiers may help in the treatment of NPC1.

Glycosylation is a major component of the post-translation process, conferring stability, ligand recognition, and signaling on its targets. It is evident from previous studies that glycosylation plays a role in NPC, but to what extent, and how it contributes in vivo, is not fully understood. Reduced O-linked glycosylation in human NPC1 fibroblasts reduces cholesterol storage and increases cholesterol efflux from these cells [26], suggesting that O-linked glycosylation in general, and specifically in the glycocalyx structure in the lysosome, may modify NPC disease progression. Furthermore, in the context of NPC1, β1,3-galactosyltransferase (β3GalT5) was identified as a possible disease modifier, as it is differentially expressed in the lobules of the cerebellum [27] and may contribute to Purkinje neuron survival in the posterior lobules of *Npc1^−/−^* mice. Our recent studies reported an approximate five-fold increase in levels of a hyperglycosylated form of LAMP1, exhibiting complex N-glycosylation, on the surface of activated microglia in the cerebellum of NPC1 mice [22], indicating a possible functional role of this form of LAMP1 during neuroinflammation in NPC1 disease. In addition, the presence or absence of complex N-linked glycosylation of NPC1 itself, determined by endoglycosidase H (EndoH) digestion, is routinely used to measure the trafficking efficacy of NPC1 and its mutants through the cell [28].

In our present study, we used a mouse model to investigate the potential role and significance of complex N-glycosylation in the context of NPC1. To that end, we chose to use mice devoid of MGAT5 [20], a Golgi-resident glycosyl transferase important in the addition of β1,6GlcNAc to produce the quatra-antennary branch at the base of the complex N-linked glycosylation structure. *Mgat5^−/−^* mice exhibit greatly reduced complex N-glycosylation (Figure S5), but are viable and appear to be phenotypically normal. When crossed with our *Npc1^−/−^* mice to generate double knock-out mutants, we found NPC1 disease progression was potentiated. In the absence of MGAT5, *Npc1^−/−^* mice exhibited earlier liver disease (Figure 6), a 1–2 week earlier progression of the disease phenotype, and increased beam walk latency (Figure 1C,D). The *Mgat5^−/−^:Npc1^−/−^* mice also had a modest but significant reduction in lifespan relative to *Mgat5^+/+^:Npc1^−/−^* mice (Figure 2). Notably, the *Mgat5^−/−^:Npc1^−/−^* mice were smaller than the single mutant *Npc1^−/−^* mice (Figure 1A,B and Figure S1) but appeared to be mobile and exhibited normal behavior prior to the onset of the neurological disease. It is possible that the reduced weight is secondary to the more severe liver disease phenotype in the *Mgat5^−/−^:Npc1^−/−^* mice. However, *Mgat5^−/−^* mice have been reported to be more susceptible to inflammatory bowel disease [29], and *MGAT5* variants in patients have been implicated in ulcerative colitis [30]; thus, in combination with intestinal inflammation described in *Npc1^−/−^* mice [31], the combined deficiencies may have exacerbated this problem, resulting in a runted state of the *Mgat5^−/−^:Npc1^−/−^* mice. In this respect, Crohn’s disease, a form of inflammatory bowel disease has been reported in some patients with NPC1 [32,33], suggesting a possible link between N-glycosylation and Crohn’s disease in NPC1. It is difficult to know from the current study whether liver disease is the cause of a shorter lifespan. However, loss of Purkinje neurons and the increased composite disease score as a measure of the neurological phenotype would suggest neurodegeneration plays a significant role.

Analysis of cerebellar tissue showed reduced calbindin protein in the cerebellar extracts (Figure 3 and Figure S3) and reduced Purkinje neuron staining in sagittal cerebellar sections (Figure 4), suggesting an increase in neurodegeneration. In support of this, we observed an apparent increase in GFAP staining in the *Mgat5^−/−^:Npc1^−/−^* mice by Western blot and immunofluorescence (IF) (Figure 3 and Figure 4), indicative of increased astrogliosis and neuroinflammation. In light of our previous results showing increased complex N-glycosylated LAMP1 on microglia of *Npc1^−/−^* mice [22], it is possible that the reduced complex N-glycosylation of LAMP1 in the *Mgat5^−/−^:Npc1^−/−^* mice (Figure 3) on the activated microglia in the cerebellum may contribute to the inflammatory process. Indeed, the more ameboid shape of the microglia, as an indication of activation, in the grey matter in the hindbrain of *Mgat5^−/−^:Npc1^−/−^* mice (Figure 5) further supports this notion. The presence of LAMP1 on the surface of cells may play a role in the migratory or invasive functions of these cells, including those of metastatic cancer cells [20,34] and immune response cells [21]. However, its specific role in microglia function is unknown. The reduced Purkinje neuron levels in 5–6-week-old *Mgat5^−/−^:Npc1^−/−^* mice (Figure S3) is fully consistent with the reduced motor coordination observed in the beam walk test and increased disease severity scores (Figure 1C,D).

A genome wide association study (GWAS) [23,35] identified two SNPs in *MGAT5* associated with disease severity in individuals with multiple sclerosis (MS), an immune system disease that attacks the protective myelin sheath surrounding neurons. In addition, a recent report has identified compound heterozygous mutations in *NPC1* in a patient diagnosed with Pelizaeus–Merzbacher-like disease (PMLD), a hypomyelination defect resulting in ataxia and progressive motor dysfunction, among other symptoms [36]. Given the neurological dysfunction and neuroinflammation observed in NPC1 patients, it was of interest to determine if these SNPs involved in complex N-glycosylation are present in our cohort of NPC1 patients and correlate with disease severity. SNP analysis of 111 NPC1 patient DNA samples found both SNPs at frequencies expected in the general population, in addition to the subpopulation of patients identified as being of European decent. Furthermore, metrics reported for disease onset and severity were not statistically different between the different genotypes (Figure S4). Thus, our analysis indicated no obvious association of these two *MGAT5* SNPs with the NPC1 disease phenotype. However, given the diverse genetic and phenotypic heterogeneity found in humans versus the defined background of the *Mgat5^−/−^:Npc1^−/−^* mice (C57BL6/SV129/Balb/c), it is possible that the role of complex N-glycosylation in NPC1 patients is masked. Potential future studies looking at the burden of variants in genes involved in complex N-glycosylation may be possible when sufficient genomic and exomic data become available.

## 4. Materials and Methods

### 4.1. Animal Maintenance

All animal work was approved by the *Eunice Kennedy Shriver* National Institute of Child Health and Human Development (NICHD) Animal Care and Use Committee (Protocol #18-002). Heterozygous *Npc1^+/−^* mice (BALB/cNctr-*Npc1^m1N^*/J) and B6.129-*Mgat5^tm1Jwd^*/J (*Mgat5^−/−^)* were purchased from The Jackson Laboratory (Bar Harbor, ME, USA). *Npc1^+/−^* mice were initially crossed with *Mgat5^−/−^* mice to obtain double heterozygous mutants (*Npc1^+/−^:Mgat5^+/−^*). These mice were then inter-crossed to generate double mutant mice (*Npc1^−/−^:Mgat5^−/−^*) and the corresponding control mice. To increase double mutant generation, *Npc1^+/^^−^:Mgat5^−/−^* males were used to cross with *Npc1^+/^^−^:Mgat5^+/^^−^* females. *Mgat5^−/−^* females were not used for breeding because information from The Jackson Laboratory indicated that they were poor mothers, as reported by the depositor of the mice (https://www.jax.org/strain/006335, accessed on 20 April 2022). Pups were weaned 3 weeks post-birth and subsequently had free access to food and water. Genotypes were confirmed by PCR using DNA extracted from ear punches and the genotyping protocol reported by The Jackson Laboratory (Protocol ID = 22784). Lectin binding studies confirmed the lack of complex-N-linked glycosylation in the *Mgat5^−/−^* mice (Figure S5), consistent with the original report describing these mice [20]. For survival studies, a humane end-of-life timepoint was determined in conjunction with the animal facility veterinarian by assessing severity of motor impairment and weight loss, at which point the mice were euthanized and tissues analyzed as necessary.

### 4.2. Weight, Disease Severity Scoring, and Survival

Mice of four genotypes (*Mgat5^+/+^:Npc1^+/+^; Mgat5^−/−^:Npc1^+/+^*; *Mgat5^+/+^:Npc1^−/−^*; *Mgat5^−/−^:Npc1^−/−^*) were assessed weekly for weight and disease severity using a standardized scoring system modified for NPC [37,38]. The disease severity score consists of five components: grooming, motor function, kyphosis, hindlimb clasp, and ledge test, with each observation scored from 0–3; 0 being the least severe and 3 indicating a severely affected phenotype. Upon entry into the behavioral room, mice were allowed to acclimate to the environment for 15 min. For the assessment, a mouse was randomly selected and weighed. The mouse was then placed into a new, empty cage and visually assessed for grooming, motor function (which included gait, tremor, and rearing), and kyphosis. The mouse was then assessed for the hindlimb clasp and immediately afterwards for the ledge test. For the ledge test, mice were placed on the ledge of the cage and assessed for their ability to walk on the ledge and round a corner with coordinated use of hindlimbs, proper balance, and ability to dismount into the cage effortlessly. A composite disease severity score was calculated by adding the scores for each assessment. Evaluations were performed by one or two observers, and mouse identification was revealed post-assessment. For survival studies, mice from the four genotypes were tracked for up to 12 weeks, and day of death was recorded when the humane endpoint was reached.

### 4.3. Beam Walk Motor Coordination Assessment

Mice were assessed for motor coordination impairment using a beam walk test. The apparatus consisted of a 70 cm long rectangular beam placed horizontally in custom made holders on either end. The beam was 1 cm wide at the start, decreasing to 0.5 cm at the end, and was set at 45 cm above the table. Layers of large bubble wrap were placed under the entire beam and surrounding area to catch any mice if they fell. Located at the end of the beam was a black box with an opening. Before testing, mice were acclimatized to the behavior room for 15 min. Individual mice were first placed in the black box with the opening covered for 1 min, to allow them to become accustomed to the box. Mice were then placed at the starting point of the beam (front paws at 30 inch mark facing the box) and the timer started; the timer was stopped when the mouse traversed the beam and entered the black box (i.e., when the nose reached the entrance). Upon finishing, mice were allowed to rest in the box with the opening covered for 30 s before starting a second time trial. A total of three trials were completed and the average time was calculated. A time limit of 60 s was set; if a mouse was unable to complete the walk or fell off the beam, a score of 60 s was assigned. The apparatus was cleaned between each mouse tested.

### 4.4. Protein Extraction and Western Blots

During this study, mice were euthanized at specific ages and tissues collected. Mice were euthanized by CO_2_ inhalation and blood obtained by cardiac puncture to obtain serum (see below). Sections of the right medial lobe of the liver were saved; in addition, the whole cerebellum was dissected and stored at −20 °C. Cerebellar and liver proteins were extracted using T-PER^TM^ lysis buffer (tissue protein extraction reagent, Thermo Fisher Scientific, Waltham, MA, USA) supplemented with 0.2% Triton X-100 and 2X Halt^TM^ protease inhibitors (Thermo Fisher Scientific). Briefly, tissues stored at −20 °C were thawed by addition of 400 μL ice-cold lysis buffer and homogenized with a handheld electrical homogenizer and plastic pestles designed to fit Eppendorf tubes (four 30 s applications). The whole cerebellum was used, and fragments of the liver were used. The homogenates were incubated on ice for 10 min, followed by a final application of the homogenizer for 30 s. The homogenates were then centrifuged at 20,000× *g* for 20 min at 4 °C. The supernatant was saved as the soluble extract of the tissues, and protein content assayed using the Bradford protein assay (Biorad, Hercules, CA, USA). For Western blot analysis, protein content in the range of 5–60 μg total protein per lane was analyzed. Primary antibodies used were rabbit monoclonal anti-LAMP1 (Cell Signaling Technology, Danvers, MA, USA, 1:2000), chicken anti-GFAP (Novus, Centennial, CO 80112, USA, 1:2000), mouse anti-calbindin (Sigma-Aldrich, St. Louis, MO, USA, 1:2000), mouse anti-CD11b (Abcam, Waltham MA, USA, 1:1000), mouse anti-actin (Sigma-Aldrich, 1:5000), mouse anti-HSP70 (BD Biosciences, Franklin Lakes, NJ, USA, 1:2000). Secondary antibodies used were IRDye anti-rabbit, anti-mouse, and anti-chicken 680 or 800 (1:10,000, LI-COR, Lincoln, NB, USA), and scanned on an Odyssey CLX infrared scanner, (LI-COR, Lincoln, NB, USA).

### 4.5. Immunohistochemical Analysis of Cerebellar Tissue

Mice were euthanized as described above at 7 weeks of age, and transcardially perfused with phosphate buffered saline (PBS). The whole brain was dissected immediately and fixed in 4% paraformaldehyde (PFA) in PBS, pH 7.4, 4 °C, for 24 h, followed by cryoprotection in 30% sucrose until sectioning. Brains were sectioned parasagitally (20 mm) in a cryostat, and sections were collected in PBS containing 0.25% Triton X-100. The floating sections were blocked for 30 min in 10% normal goat serum in PBS, and then incubated overnight at 4 °C with rabbit anti-calbindin (1:400; Cell Signaling, Danvers, MA, USA), chicken anti-GFAP (1:1000, Novus), and rabbit anti-Iba1 (1:500, Wako Pure Chemicals, Richmond, VA, USA) in 0.25% Triton X-100/PBS. After washing, the sections were incubated with appropriate host-specific secondary antibodies coupled to either Alexa 594 and Alexa 488 (1:1000, Thermo Fisher Scientific, Waltham, MA, USA). Nuclei were stained with Hoechst 33342, trihydrochloride, and trihydrate I (1:1000, Invitrogen, Carlsbad, CA, USA). Images were taken on a Zeiss Axio Observer Z1 microscope (Zeiss, Germany).

### 4.6. Serum Liver Enzyme Analyses

To assess liver function, mice were euthanized by CO_2_ and blood obtained by cardiac puncture. The samples were allowed to coagulate at room temperature for 45 min and were then centrifuged for 10 min at 2500 rpm, 4 °C. The serum was stored at −20 °C until sent for analysis. Sera from control and affected mice (5–6 weeks of age) were sent to the Department of Laboratory Medicine, Clinical Center, NIH for hepatic panel screening. The liver enzymes assayed were alkaline phosphatase (AP), alanine transaminase (ALT), and aspartate transaminase (AST), using the following Roche Diagnostics reagent kits; AP #03333752 190; ALT #20764957 322; AST #20764949 322. All assays were analyzed by Roche/Hitachi cobas c 311/501 analyzers (Roche Diagnostics GmbH, Mannheim, Germany).

### 4.7. MGAT5 Single Nucleotide Polymorphism (SNP) Analysis and NPC1 Disease Severity

Human studies were performed as part of a Natural History/Observational study (NCT00344331) approved by both an NICHD and an NIH Institutional Review Board. Written consent was obtained from participants or guardians. Assent was obtained when applicable. Two *MGAT5* SNPs (rs4953911 (T > A), rs3814022 (C > G)) have been shown to be associated with multiple sclerosis (MS) [23,35], potentially due to reduced complex N-linked glycosylation [19]. To determine if individuals with NPC1 carry these SNPs, we performed whole genome sequencing (NIST, Gaithersburg, MD, USA) and SNP genotyping (Transnetyx, Cordova, TN, USA) for 111 individuals with NPC1 enrolled in the natural history study (NCT00344331). Data were compared to the Allele Frequency Aggregator (ALFA) frequency table within the NCBI dbSNP website (https://www.ncbi.nlm.nih.gov/snp/, accessed on 20 April 2022). Age at disease onset, disease severity score at baseline, age-adjusted disease severity score, and age at first neurological symptom were then compared between genotype groups.

### 4.8. Statistical Analysis

All statistical analyses were performed in Prism software *ver.* 8.4.6 or higher (GraphPad, San Diego, CA, USA). For the survival curve analysis, the log-rank (Mantel–Cox) test was used. Serum liver enzyme analysis was by one-way ANOVA followed by multiple comparisons test. The Western blot analysis of calbindin levels was performed with an unpaired two-tailed *t* test. For *MGAT5* SNP analysis, allele frequency for each SNP in our cohort was calculated. Gene frequency was subsequently determined using the Hardy–Weinberg equation. Chi-square analysis was performed in Prism software as a contingency table analysis between observed and expected gene frequencies. In all cases noted, significance was set to *p* < 0.05 (*), <0.01 (**), <0.001 (***).

## Data Availability

Not applicable.

## Supplementary Materials

The following supporting information can be downloaded at: https://www.mdpi.com/article/10.3390/ijms23095082/s1.

## Abbreviations

NPC1, Niemann–Pick disease type C1; LAMP1, lysosome-associated membrane protein 1; MGAT5, β1,6N-acetylglucosaminyltransferase V; GFAP, glial fibrillar acidic protein; SNP, single nucleotide polymorphism.
