# Supplementary material for: Complex N-Linked Glycosylation: A Potential Modifier of Niemann–Pick Disease, Type C1 Pathology
Source: Int J Mol Sci. 2022 May 3;23(9):5082. doi: 10.3390/ijms23095082 (PMC9103943; doi:10.3390/ijms23095082)
Supplement: Supplementary file 1 [file ijms-23-05082-s001.zip › ijms-1666192(8)(2) updated.pdf]

The *Mgat5*$^{-/-}$*/Npc1*$^{-/-}$ mice are smaller than the *Mgat5*$^{+/+}$*/Npc1*$^{-/-}$ mice

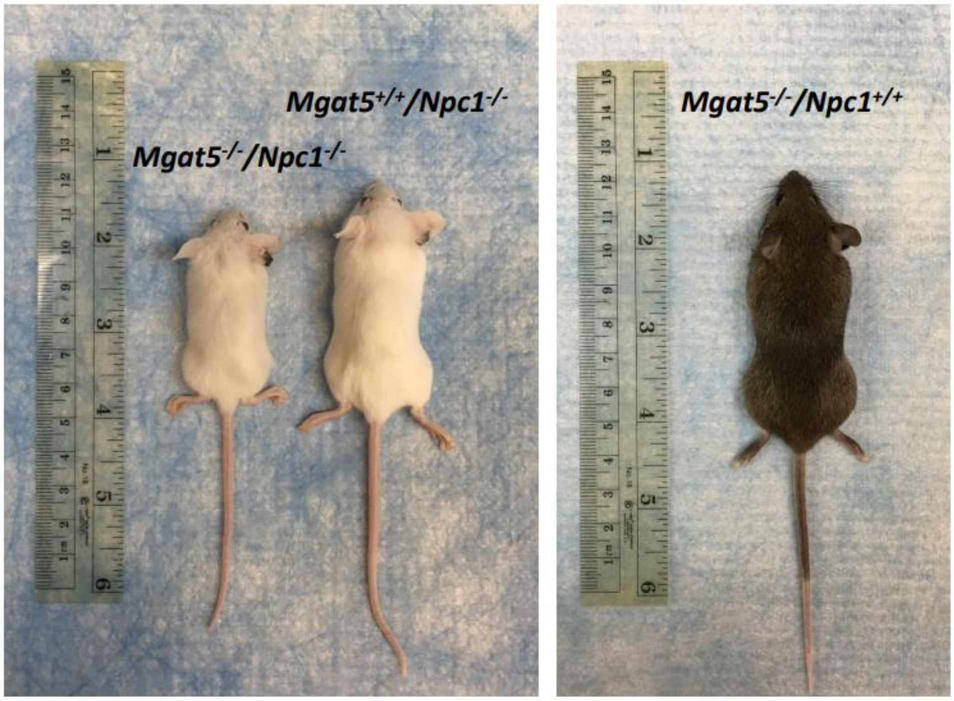

7-week-old mutant mice

Supplementary Figure S1. Photographs of 7-week-old female mutant mice. The double mutant *Mgat5*$^{-/-}$*:Npc1*$^{-/-}$ mice are smaller than the single mutant *Mgat5*$^{+/+}$*:Npc1*$^{-/-}$ mice. *Mgat5*$^{+/+}$*:Npc1*$^{-/-}$ mice are smaller than both the control mice; *Mgat5*$^{-/-}$*:Npc1*$^{+/+}$ (shown) and *Mgat5*$^{+/+}$*:Npc1*$^{+/+}$ (not shown). Similar observations were seen for the male mice (not shown).

Western blot analysis of LAMP1 in liver extracts from control and mutant mice

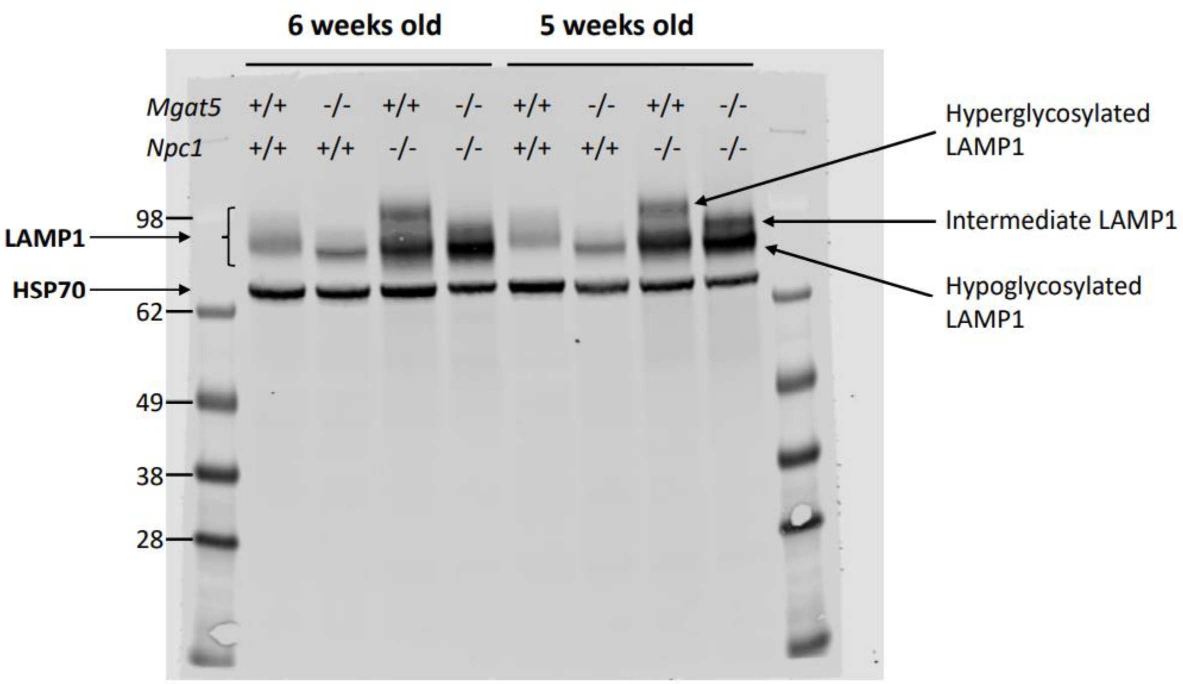

Supplementary Figure S2. Western blot analysis of LAMP1 in liver extracts from control and mutant mice. Total soluble protein (~5 ug) from the liver of 5- and 6-week-old mice were analyzed by western blot for LAMP1. Note the size reduction of the hyperglycosylated LAMP1 to an intermediate size in both *Npc1* control and mutant mice in the absence of MGAT5. Heat shock protein 70 (HSP70) was used as protein loading control.

Figure S3

Western blot analysis of Calbindin levels in Cerebellar extracts

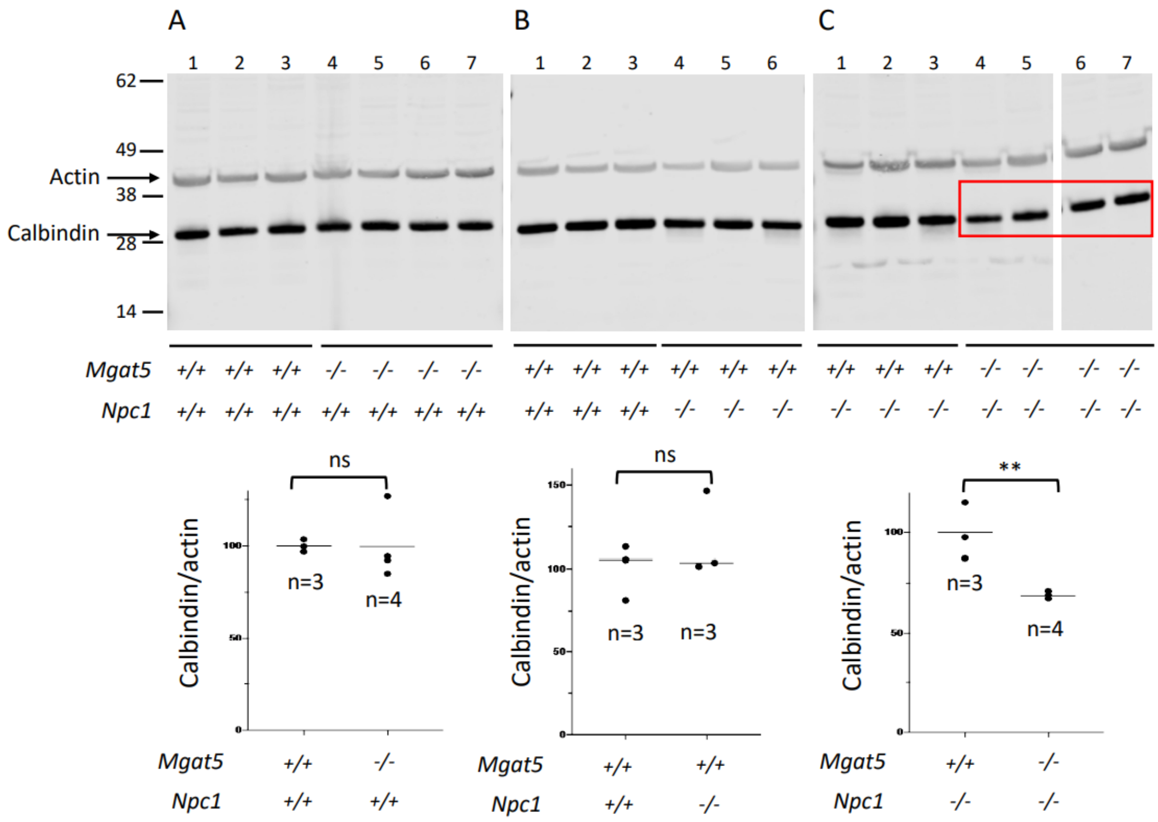

Supplementary Figure S3. Quantification of Calbindin in mouse cerebellar extracts. Total soluble protein (20 ug) from whole cerebella of 6-week-old mice were analyzed by western blot for calbindin and actin. The immunoblots were scanned and analyzed by the LiCor Odyssey system. Band intensity of Calbindin was normalized by actin intensity for the same lane and analyzed by Prism (version 8.3.6). Note the reduced levels of Calbindin in the double mutant *Mgat5*$^{-/-}$*:Npc1*$^{-/-}$ mice (**C**) compared to the the single mutant *Mgat5*$^{+/+}$*:Npc1*$^{-/-}$ mice at this age. No significant differences in levels of calbindin were observed in the other mouse comparisons (**A, B**).

NPC1 disease metrics as a function of four *MGAT5* SNP genotypes

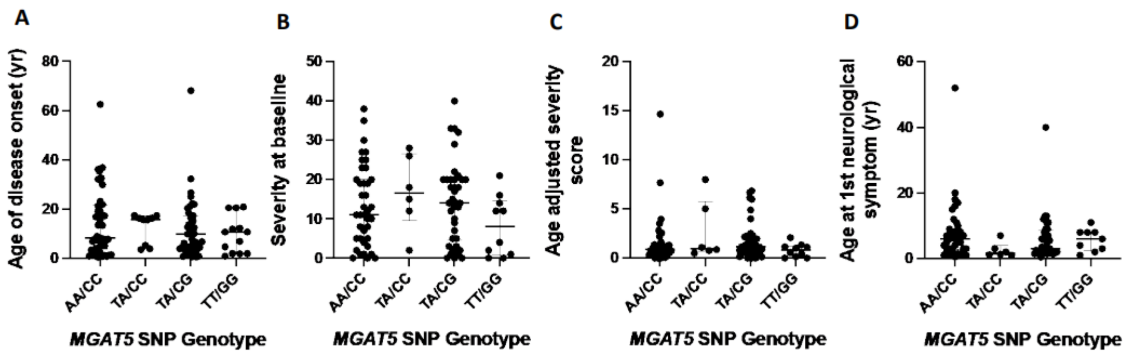

Supplementary Figure S4. Graphical analyses of NPC1 disease metrics as a function of four *MGAT5* SNP genotypes. *MGAT5* SNP sequence of 101 NPC1 patients were grouped into the 4 major groups obtained and their scores for age of disease onset (**A**), disease severity score at baseline (**B**), age adjusted disease severity score (**C**) and age at first neurological symptom (**D**) were plotted. In all analyses, no significant differences were observed between groups. Bars indicate median with the interquartile range.

Phaseolus Vulgaris Leucoagglutinin (PHA-L) staining of cerebellar samples

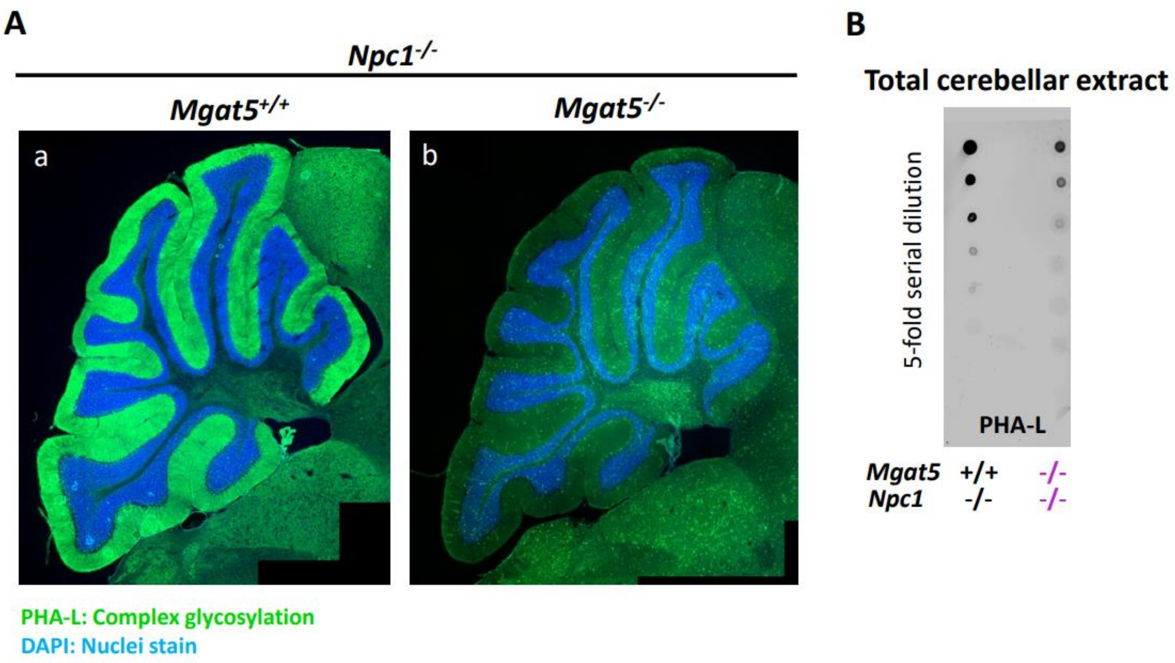

Supplementary Figure S5. Lectin binding analysis of mouse cerebellar tissue. Biotinylated Phaseolus Vulgaris Leucoagglutinin (PHA-L) was used to probe mouse cerebellar sections and total cerebellar soluble protein extracts of *Npc1* mutant mice by histochemistry (**A**) or dot blot (**B**). Bound lectin was visualized by FITC- or horseradish peroxidase-labelled streptavidin. The reduced signal of PHA-L in $Mgat5^{-/-}$:$Npc1^{-/-}$ mice confirms the functional absence of MGAT5 in these mice.
